# From Hypoxia to Buoyancy: Serotonin and Oxygen Deprivation Rewire Neutrophils

**DOI:** 10.21203/rs.3.rs-5349113/v1

**Published:** 2024-11-19

**Authors:** Luz P. Blanco, Jorge Romo-Tena, Xinghao Wang, Yaima L. Lightfoot, Mariana J. Kaplan

**Affiliations:** National Institutes of Health; National Institutes of Health; National Institutes of Health; National Institutes of Health; National Institutes of Health

**Keywords:** Neutrophils, hypoxia, serotonin, tryptamine, systemic lupus erythematosus, NETosis

## Abstract

Herein, we describe a method to purify higher buoyancy neutrophils *in vitro* after exposure of whole blood to brief hypoxia and reoxygenation in combination with platelet-derived serotonin. These higher buoyancy neutrophils display enhanced ability to form neutrophil extracellular traps and increment the tryptamine-protein adducts. Similar changes are identified in neutrophils isolated from patients with systemic lupus erythematosus. These results suggest that neutrophils may be rewired in tissues under conditions of hypoxia-reperfusion such as those seen in chronic inflammatory conditions.

## INTRODUCTION

Neutrophils isolated from patients with chronic inflammatory and/or autoimmune conditions, including systemic lupus erythematosus (SLE), are often exposed to oxidative and hypoxic environments. These conditions may arise from poor circulation and tissue oxygenation, which are common in vasculopathies associated with these diseases [1–3]. Neutrophils, in response to inflammation, produce reactive oxygen species (ROS) through oxidative bursts, consuming oxygen and promoting a state known as inflammatory hypoxia [4]. In this work we describe that, after brief hypoxia-reoxygenation exposure neutrophils, enhance their ability to NET and acquire higher buoyancy or lower density like-phenotype. Furthermore, we found that similar findings are observed in neutrophils purified from patients with autoimmune diseases.

## METHODS AND MATERIALS

### Human Samples

Venous blood from SLE or healthy control females followed at the Clinical Center (NIH) was drawn onto heparinized tubes All individuals signed informed consent in respective IRB-approved protocols (NIH 94-AR-0066). The blood remained at room temperature for no more than 2 h before further use.

#### Isolation of PBMCs, Plasma, Neutrophils, *Bona fide* LDG, and NETs,

A 30 ml volume of whole blood was carefully added onto 20 ml Ficoll-Paque solution (catalog #: 45-001-755, Fisher Scientific, Waltham, MA) and centrifuged for 25 min at 1440 rpm with no break or acceleration. PBMCs were harvested from the interphase layer and plasma was collected from the upper clear yellow layer. Plasma was filtered through 0.2 u disposable filters to obtain the platelet-free plasma used in some experiments. Neutrophils were isolated from the sedimented red blood cells pellet by resuspending them in 20% dextran (volume/volume) for 20 min at room temperature. After that time, neutrophils were centrifuged from the clear fraction and the red blood cell sediment discarded. The supernatant was further centrifuged at 1,600 rpm for 5 min, the cellular pellets were then resuspended in 20 ml 0.2% saline solution and incubated for 1 min. Saline solution 1.5% (30 ml) was added and the tubes were again centrifuged. The neutrophil pellet was washed one time in PBS and resuspended in RPMI1640 (catalog #: 11835030, Thermo Fisher Scientific, Waltham, MA) for further use. *Bona fide* LDGs were isolated from SLE PBMC by negative selection with magnetic beads as previously described [5]. Spontaneously generated NETs were isolated as previously described using DNase I (RNase free) for their detachment (catalog #: 4716728001, Sigma Aldrich (originally Roche), St. Louis, MO) [2].

### Generation of Higher Buoyancy Neutrophils

Higher buoyancy neutrophils were generated either after treatment of whole blood or by using 10 × 10^6 NDGs in 5 ml of RPMI1640 containing 1:4 autologous plasma. The treatments of hypoxia gradient were 30 min for whole blood or 1.5 h for the isolated NDGs, using the GasPack EZ pouch system (catalog #: B260684, Fisher Scientific (BD original brand), Waltham, MA). In other experiments exogenous serotonin (catalog #: H9523, Sigma Aldrich, St. Louis, MO) was added directly to NDGs at 1.75 uM final concentration in RPMI1640 before the hypoxia treatment for 1.5 h.

### Flow Cytometry

**Figure 1:**
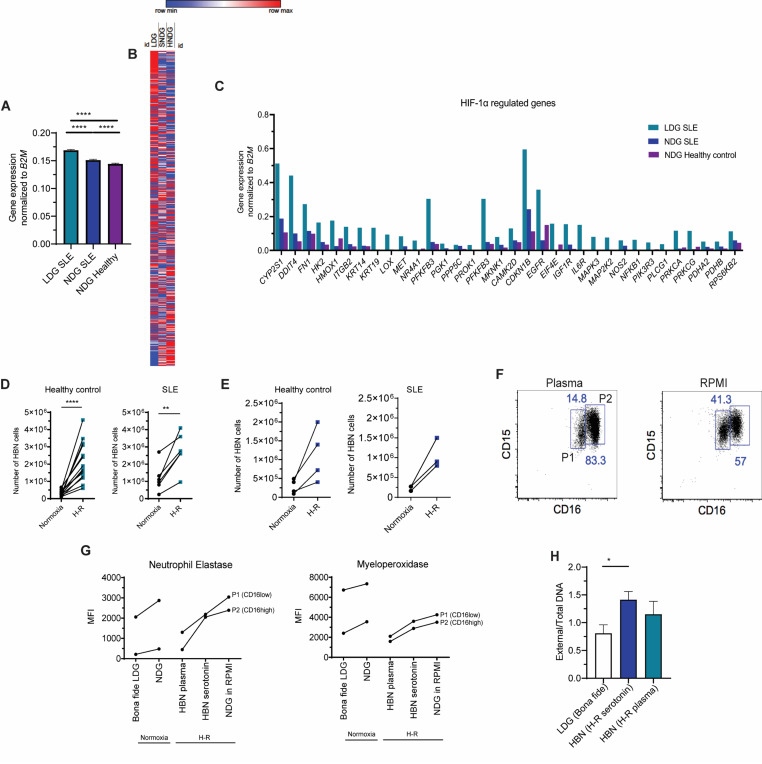
Characterization of hypoxia-induced gene expression in LDGs and generation of HBN cells *in vitro* **A.** Transcriptomic analysis of hypoxia regulated genes using previously published transcriptomic data [16]. **B.** Heat map of the transcriptomic analyses of three different cell types analyzed in A prepared using Morpheus online tool (https://software.broadinstitute.org/morpheus). **C.** Specific HIF-1 alpha target genes with differential expression for the three cell types analyzed using DAVID online tool (https://david.ncifcrf.gov/). Hypoxia regulated genes (n=15,158) analyzed in these figures were gathered from the online database (integrative database of expression dynamics of proteins in response to hypoxia: http://ihypoxia.omicsbio.info/). **D.** Number of high buoyancy (HBN) cells generated after treatment of 10^6 NDGs isolated as previously described [5] from healthy donors or from SLE patients (see Supplementary information for detailed materials and methods). Cells were cultured in RPMI containing autologous plasma (1:4) and quantified 1.5 h after either normoxia (black) or H-R treatment (blue). **E.** Similar to D, results represent the number of high buoyancy neutrophils generated after culturing healthy control or SLE NDGs in RPMI containing exogenously added serotonin (1.75 uM) after 1.5 hours of either normoxia (black) or H-R (blue). **F.** Representative results of flow cytometry analysis of SLE NDGs exposed to H-R for 1.5 h in either autologous plasma (1:4 in RPMI) or RPMI alone. **G.** Neutrophil degranulation was quantified by flow cytometry, determining the intracellular mean fluorescent intensity of neutrophil elastase and myeloperoxidase (MPO) in neutrophils isolated from 2 SLE patients as well as for the cells characterized in F. (H) NETosis was quantified *in vitro* by fluorometric plate assay, as previously described [17] of *bona fide* SLE LDGs (isolated by negative selection as previously described [5]), or HBN generated by treatment with H-R plus serotonin (1.75 uM, royal blue) or H-R in autologous plasma (blue) from 2 SLE donors. Data was analyzed using Mann Whitney U test. *:P<0.05.

Flow cytometry to analyze LDGs and hypoxia-generated high buoyancy neutrophils (LDG-like cells) in PBMC for Fig. 1, was done as previously described [1]. IDT-307 (catalog #: SLM0756, Sigma Aldrich, St. Louis, MO) was used to assess serotonin transporter functionality at 1 uM final concentration and after 1 h incubation at 37°C. Granule content analysis of LDGs and NDGs was done by incubating cells with human FcR blocking reagent (catalog #: 130-059-901, Miltenyi) for 10 minutes at 4°C prior to surface staining with CD15-PE/Cy7 and CD16-PerCP/Cy5.5 (catalog #:323029 and 302027, Biolegend) for 30 minutes at 4°C. Cells were then fixed and permeabilized with BD Cytofix/Cytoperm^™^ Fixation/ Permeabilization Solution Kit (catalog #: BDB554714, BD Biosciences) for 20 minutes at room temperature per manufacturer’s instructions. Cells were then stained with myeloperoxidase (MPO) (catalog #: 11-1299-42, Thermo Fisher Scientific) and neutrophil elastase (NE)-FITC (catalog #: orb3644, Biorbyt) antibodies for 1 hour at room temperature. FACS analysis was performed with a BD LSRFortessa^™^ Cell Analyzer, and data was analyzed in FlowJo.

### Fluorometry

Experiments to assess serotonin transporter function in neutrophils were performed by plate assay (96 wells) using isolated neutrophils (1×10^6 /ml) resuspended in RPMI or autologous plasma with or without platelets (after filtering through a 0.2 u disposable filter). The serotonin reporter dye IDT-307 (ex/em: 440/520 nm) was added at 1 uM final concentration and, following incubation for 1 h at 37°C fluorescence measurements were done in a BIOTEK Synergy HTX multiplate reader (Agilent).

### Immunofluorescence

Tryptamine adducts contained in granulocyte proteins were quantified in adherent cells in cover chambers (catalog #: UX-01838-12, Fisher Scientific, Waltham, MA). The cells were fixed in 4% paraformaldehyde for 30 min, washed in PBS, permeabilized in 0.2% Triton X-100 for 10 min, washed, and blocked for 20 min in 0.2% gelatin. Washed cells in PBS were then treated with polyclonal rabbit antibody against conjugated tryptamine, (catalog #: ab8885; 1:100; Abcam, Cambridge, UK currently discontinued but similar to a commercially available rabbit polyclonal antibody against tryptamine catalog: DPAB1805 from Creative Diagnostics) overnight at 4°C, washed in PBS and treated with a secondary FITC-donkey anti rabbit antibody 1:4 (catalog #: A31572, Thermo Fisher) and 1:1000 Hoechst 33342 (catalog #: H3570, Thermo Fisher Scientific) for 1 h at room temperature. Cells were washed and mounted with ProLong gold (catalog #: P36930, Thermo Fischer Scientific). Cells were imaged using a Zeiss Laser Confocal LSM780 microscope and the ZEN 2.6 software.

### Western Blot

Cells were lysed in RIPA buffer containing 150 mM NaCl, 1.0% IGEPAL CA-630, 0.5% sodium deoxycholate, 0.1% SDS, 50 mM Tris, pH 8.0 (catalog #: R0278; Sigma-Aldrich, St. Louis, MO) with protease inhibitor mini tablets (AEBSF, aprotinin, bestatin, E-64, leupeptin, and pepstatin A) (catalog #: A32955; Thermo Fisher Scientific, Waltham, MA), and placed at 4°C on a rotator for approximately 30 min. Lysates were centrifuged at 20,817 g for 10 minutes at 4°C, and the supernatants were transferred to a new Eppendorf tube. Protein content in cell lysates or NETs was quantified using a BCA protein assay kit (catalog #: 23225; Invitrogen/Thermo Fisher Scientific, Waltham, MA). Equivalent amounts of protein were resolved in a 4–12% gradient bis-tris gel (catalog #: NP0335BOX, Invitrogen/Thermo Fisher Scientific, Waltham, MA) in the presence or absence of β-mercaptoethanol. Proteins were immobilized onto a nitrocellulose membrane (Invitrogen) and blocked with 10% bovine serum albumin for 30 minutes at room temperature. Thereafter, the membrane was incubated with polyclonal rabbit antibody against conjugated tryptamine, (catalog #: ab8885; 1:100; Abcam, Cambridge, UK), rabbit anti-histone 3 (catalog #: ab70550; 1:1000; Abcam, Cambridge, UK), or mouse anti-GAPDH (catalog #: AM4300; 1:1000; Invitrogen/Thermo Fisher Scientific, Waltham, MA) overnight at 4°C. After 3 washes with PBS–Tween, the membrane was probed with secondary IRDye800 goat anti-rabbit antibodies or IRDye800 goat anti-mouse (1:10,000; Li-Cor Biosciences) for 1 h at room temperature. Proteins were detected using a Li-Cor Biosciences Odyssey Infrared Imaging System, in accordance with the manufacturer’s instructions.

### Statistical Analysis

Data is expressed in average ± SEM with at least three repeats per experiment. The analysis was done using GraphPad Prism software and the significant P values are indicated in the figure legends.

## RESUTS AND DISCUSSION

To investigate how hypoxia affects neutrophils, especially under proinflammatory conditions, we studied the transcriptomic profiles of neutrophils isolated from SLE patients. We specifically focused on a subset called low-density granulocytes (LDGs), which are known to be proinflammatory [6]. Our analysis revealed that SLE LDGs display a hypoxic gene expression signature compared to normal-density neutrophils (NDGs) from both SLE patients and healthy individuals. This includes increased expression of HIF-1 alpha target genes, a key regulator of the body’s hypoxic response (Fig. 1A-C).

Previous research has shown that hypoxia influences neutrophil biology, including reduced apoptosis and increased degranulation and autophagy [7–9]. We demonstrated that incubating neutrophils under hypoxic conditions (90 minutes) followed by reoxygenation (H-R) generates high-buoyancy (HBN), LDG-like cells. This process requires the presence of autologous platelet-rich plasma (1:4 dilution). To identify the component in plasma necessary for generating HBN cells after hypoxia-reoxygenation (H-R), we conducted a series of experiments that revealed that the key factor was a small, non-proteinaceous molecule, which we later identified as serotonin (Fig. 1D-E). Flow cytometry analysis revealed the presence of two CD15+ neutrophil populations based on CD16 expression (P1 and P2), with the CD16^high^ subpopulation (P2) predominating under H-R conditions in plasma, indicating a shift in neutrophil phenotype (Fig. 1F).

Degranulation, measured by levels of intracellular neutrophil elastase and myeloperoxidase (MPO) staining, was elevated in hypoxia-induced HBN, comparable to *bona fide* SLE LDGs (Fig. 1G). We also found that serotonin-treated neutrophils exhibited intermediate levels of degranulation compared to other conditions. Additionally, NETosis (the formation of neutrophil extracellular traps) was significantly higher in H-R neutrophils treated with serotonin than in *bona fide* LDGs (Figure 1H).

**Figure 2:**
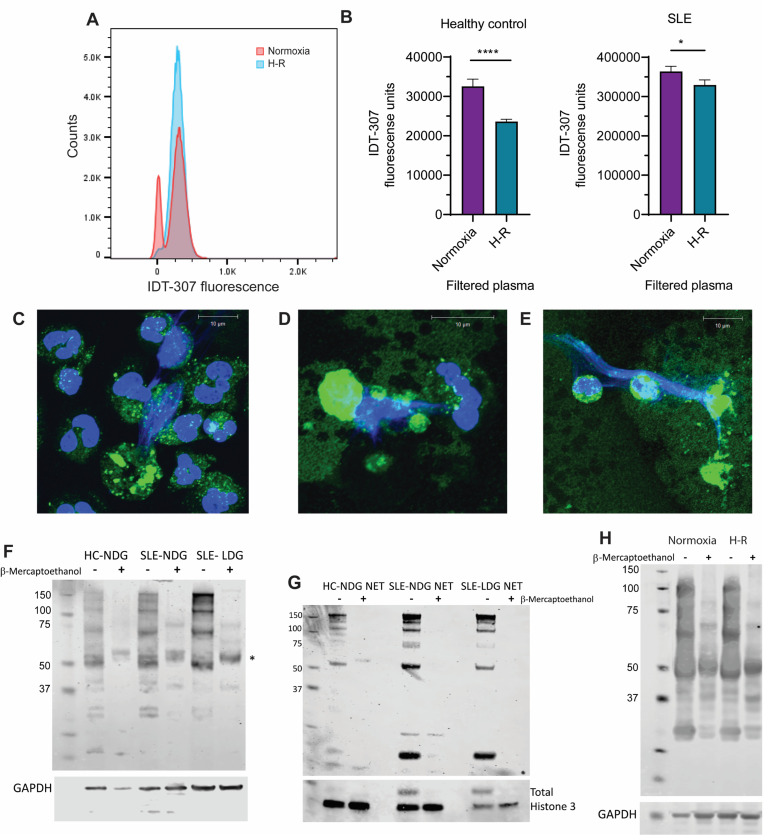
Serotonin internalization and tryptaminization induced by H-R. **A.** NDG internalization of IDT-307 (ex/em: 440/520 nm, 1 uM, 1h 37°C) measured by flow cytometry. Granulocytes were gated from healthy control peripheral blood after treatment of whole blood with either normoxia or H-R for 30 min (see Supplementary information for detailed materials and methods). **B.** Effect of filtered plasma containing platelet-derived serotonin in the IDT-307 internalization by NDGs isolated from healthy controls (n=2) or SLE patients (n=3). Shown is the average ± SEM, data analyzed using Mann Whitney U test; *: *P* <0.05; ****: *P* <0.0001. Laser confocal immunofluorescence images of lupus neutrophils stained with an antibody that recognizes melatonin and cross reacts with tryptamine adducts (green) and Hoechst (DNA, blue) showing NDG in **C** *bona fide* LDG in **D** and high-buoyancy neutrophils generated *in vitro* by H-R incubation in autologous plasma (1:4 in RPMI) for 1.5 h in **E.** Protein tryptaminization was detected with Western blot analysis using ab8885 antibody and cell types in **F** or NETs in **G** from either healthy donors (HC) or SLE patients. The asterisk indicates a protein adduct that is insensitive to β-mercaptoethanol in F. **H.** Protein tryptaminization detected by Western blot in isolated NDGs from a lupus patient after treatment of whole blood with normoxia or H-R for 30 min.

To further explore the role of serotonin, we assessed serotonin transporter activity in neutrophils using the fluorescent reporter IDT-307. Neutrophils exposed to H-R showed increased serotonin transporter activity compared to normoxic neutrophils (Figure 2A). Plasma devoid of platelets (filtered) under H-R conditions, from both SLE patients and healthy controls, also inhibited serotonin internalization by neutrophils, suggesting that hypoxia increases serotonin availability in plasma (Fig. 2B). These findings suggest that H-R enhances both serotonin levels in plasma and serotonin transporter activity in neutrophils.

SLE patients often exhibit disrupted platelet biology, with reduced serotonin levels both in plasma and within platelet dense granules [10]. Internalized serotonin may benefit most cells by serving as a precursor to melatonin, a powerful mitochondrial antioxidant [11]. However, in neutrophils laden with oxidative enzymes like MPO, serotonin oxidation may lead to harmful protein modifications [12–15]. Our data show that H-R promotes tryptaminization—a form of protein alkylation—in neutrophils, as confirmed by immunofluorescence and Western blot analysis (Fig. 2C-H).

In conclusion, our findings highlight a novel mechanism by which H-R and serotonin may alter neutrophil biology in inflammatory conditions. While these are in vitro-generated findings, it is possible that H-R plays a role in the generation of aberrant neutrophil subsets in conditions associated with hypoxia, chronic inflammation, and platelet dysfunction. Indeed, H-R appears to generate a process of neutrophil dysregulation, potentially contributing to disease pathology.

## CONCLUSION

Higher buoyancy neutrophils are generated after a brief hypoxia-reoxygenation treatment. These rewired neutrophils acquire enhanced ability to generate NETs and incremented tryptamine adducted proteins. The transformation requires platelet derived serotonin.

## Data Availability

All data and materials are available upon emailing the corresponding author.
